# Doctor and Patient

**Published:** 1888-03

**Authors:** 


**Doctor and Pat ent.** By S. Weir Mitchell, M. D., LL. D., Harv., Member of the U. S. National Academy of Science, etc. Pp. 177. J. B. Lippincott Company, publishers.

There are few men in the medical profession with talents more versatile than those of the distinguished author of this book. As a medical writer, he is highly instructive; as a lecturer, all admire his genius and clear didactic discourse; as a novelist, he has charmed many a reader, and as a teacher of popular medicine, he has few equals. To this last class of writings the present work belongs, and although it was our first intention merely to skim through it, so entertaining and instructive did we find the book, that the entire work was read. Not only will intelligent patients, for whom the work was intended, be interested in these lectures, but medical men will obtain from it many valuable hints for the treatment of their patients.

